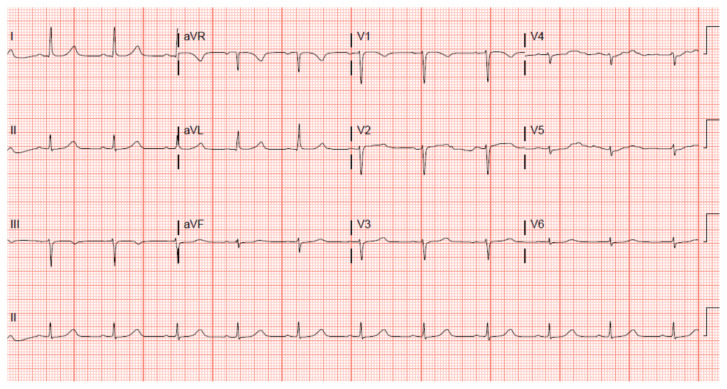# ’Cath’ It Before It’s Too Late: A Case Report of ECG Abnormalities Indicative of Acute Pathology Requiring Immediate Catheterization

**DOI:** 10.21980/J8HW7V

**Published:** 2022-07-15

**Authors:** Diane Wei, Paul Truong, Patrick Bruss

**Affiliations:** * University of Toledo College of Medicine, Department of Emergency Medicine, Toledo, OH; ^ Promedica Monroe Regional Hospital, Department of Emergency Medicine, Monroe, MI

## Abstract

Current guidelines to detect for myocardial infarction (MI) are not sufficient to triage patients requiring immediate cardiac catheterization, with at least 25% of non-ST elevation myocardial infarction (NSTEMI) patients found to have acute coronary occlusion (ACO) only on delayed catheterization, and up to 35% of perceived ST-elevation myocardial infarction (STEMI) ACOs found as false positives at catheterization. There has been a call for an integration of a new paradigm: occlusion/non-occlusion MI (OMI/NOMI). Here we discuss a 51-year-old female who presented to the emergency department with subtle electrocardiogram (ECG) abnormalities not adherent to the current MI guidelines. However, the subtle abnormalities when combined with her history and risk factors point to a high probability of a pathological amount of coronary disease that required immediate catheterization. This case report illustrates the importance in revisiting current guidelines and the need to integrate OMI/NOMI alongside current guidelines to guide decision making for immediate reperfusion needs.

**Topics:**

Occlusion, myocardial infarction, ST elevation myocardial infarction, acute coronary syndromes, electrocardiography.

## Brief introduction

Acute coronary occlusion (ACO) reperfusion decreases mortality and morbidity in patients following acute myocardial infarction (MI).^1^ The current fourth universal definition of MI focuses on the presence or absence of ST-elevation, dichotomizing the MI as either ST-elevation myocardial infarction (STEMI) or non-ST elevation myocardial infarction (NSTEMI) based on guidelines.^2^ However, it has become increasingly evident that the presence or absence of ST-elevation does not exclusively rule in or out acute coronary occlusions. The current MI guidelines have led to at least 25% of NSTEMI patients to be found to have ACO only on delayed catheterization, and up to 35% of perceived STEMI ACOs to be found as false positives at cardiac catheterization.^3^,^4^ A new paradigm shift towards the concept of occlusion/non-occlusion MI (OMI/NOMI) has proven to rapidly and noninvasively identify patients for reperfusion.^5^ This case report takes OMI/NOMI into context and the increasing importance in integrating it alongside traditional STEMI/NSTEMI guidelines to determine patients’ immediate needs for percutaneous coronary intervention. We believe the combination of cardiac risk factors, cardiac symptoms, and focal repolarization abnormalities on the electrocardiogram (ECG) are a better indication for emergent revascularization than relying solely on the amount of ST-elevation.

## Presenting concerns and clinical findings

**Figure 1:**
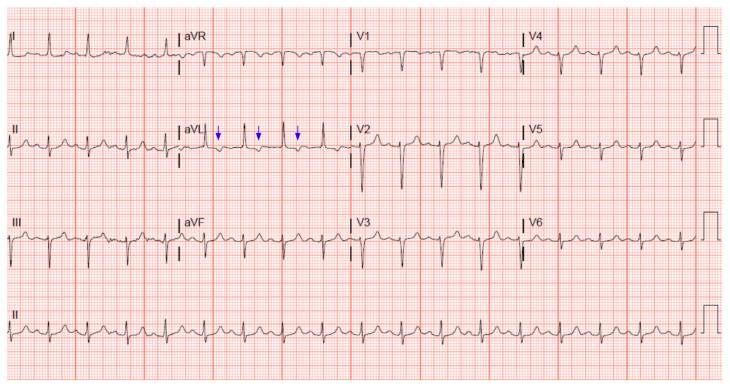


A 51-year-old female came into the emergency department complaining of vomiting along with concerns regarding recent labs that had revealed anemia. Her relevant medical history includes antitrypsin 1 deficiency and liver cirrhosis with ascites and abdominal paracentesis along with other comorbidities. She had smoked for 25 pack years before quitting back in 2019. Her family history was significant for heart disease, hypertension, and colon cancer. On review of symptoms, she did report chest pain recently with exertion but denied any active chest pain while in the emergency room while the first ECG was done (see Figure 1).

The patient’s initial troponin was negative. She had multiple other metabolic abnormalities including anemia, worsening of ascites, and liver failure and was admitted to the intensive care unit (ICU). Two days later, while in the ICU, she developed chest pain and a repeat ECG and troponin were obtained. The chest pain was a substernal pressure radiating to the back and was associated with nausea and shortness of breath. The repeat serum troponin was elevated at 1.73 ng/mL and the echocardiogram obtained showed moderate global hypokinesis. She was subsequently taken to the catheterization lab which revealed 100% occlusion of the left anterior descending artery that was stented. Unfortunately, the patient expired during her stay.

## Significant findings

**Figure 2:**
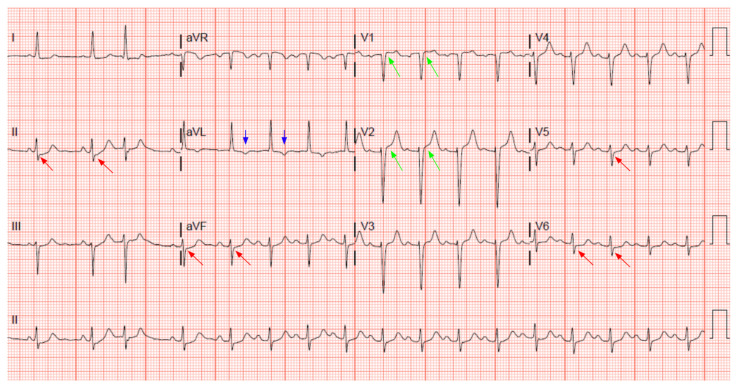


A 12 lead ECG performed at the time of emergency department (ED) admission revealed regular sinus rhythm with noted Twave inversion (blue arrows on Figure 1) in Lead aVL new when compared to an ECG performed a few months prior (see Figure 3). Two days later a second ECG was done when the patient developed acute chest pain while in the ICU (Figure 2) that showed persistent inversion in Lead aVL (blue arrows) as well as new J point deviation (JPD) in Leads II, aVF, V5 and V6; and new JPD in Leads V1 and V2 (green arrows) from her previous ECG while in the emergency department. These focal repolarization abnormalities did not qualify as an ST-elevation myocardial infarction by current guidelines.

## Discussion

A rising issue in emergency cardiology is being able to differentiate patients who have ACO from those who do not to ultimately avoid harming them from either unnecessary invasive interventions or delayed catheterization. Thus, identifying the need for catheterization laboratory efficiently and accurately is crucial. Current criteria focus on specifically defined ECG findings based on the absence or presence of 1) 1-mm ST elevation (STE) in any two contiguous leads except V2 and V3 and 2) STE in V2 and V3 on basis of age and gender where the following cut points apply: >=1.5 mm elevation in women regardless of age, >=2.5 mm in men aged less than 40 years, and >=2mm in men aged 40 years and above.^2^

The case demonstrated that this patient’s ECGs revealed no evidence that qualified as an ST-elevation MI per current guidelines. Catheterization revealed LAD (left anterior descending) occlusion, confirming the diagnosis of a type 1 myocardial infarction despite the lack of qualifying ECG criteria. However, there were subtle ECG findings to indicate occlusion was present all along, especially when considered in conjunction with her symptoms and cardiac risk factors.

An acute occlusion of the coronary vessels results in abnormalities of blood flow to a focal area of the myocardium, leading to abnormal conduction of electricity through that area of the myocardium. Classically, this results in ST elevation in contiguous leads of the ECG, which one can consider as extreme focal repolarization abnormalities. However, we believe that JPD from the PR segment, be it elevation or depression, is a more accurate finding to look for than the amount of ST elevation, especially when seen in conjunction with cardiac risk factors and symptoms. We believe this case highlights the fact that the presence of focal repolarization abnormality is a more crucial concept than the amplitude of ST elevation for the diagnosis of acute myocardial infarction, especially when these abnormalities are present in patients with symptoms and risk factors of coronary disease.

We feel the combination of symptoms with risk factors and focal repolarization abnormality represents a high pretest probability of the patient having a pathological amount of coronary disease that warrants emergent heart catheterization and that this approach may be superior to relying solely on the amount of ST elevation. In this case, T wave inversion in AVL as well as JPD from the PR segment were compelling focal repolarization abnormalities concerning for ACS. The T-wave inversion, which was new from previous ECG seen on the day of the ED visit, continued to be seen throughout successive ECGs. At the time of the acute chest pain, ECG showed new abnormal findings of JPD in Leads II, aVF, V1, V2, V5 and V6 (>1 mm JPD from the PR segment. J point deviation is often seen in ST segment abnormalities. These findings on ECGs suggest pathology.

Though these findings did not fulfill current STEMI criteria, taken together with her relevant past medical history and presentation at the time of the chest pain, they demonstrated the patient was suffering from an occlusion and had required immediate catheterization. Troponin levels and echocardiogram further supported MI, but were not immediately available. The serum troponin levels were found to be elevated five hours after the initial presentation. However, the abnormal ECG findings alongside patient risk factors should be sufficient to raise clinical suspicion even prior to echocardiogram results. A holistic approach should be taken when evaluating patients for emergent catherization, which is the central aspect of the OMI/NOMI paradigm.

Many OMI do not present with elements that meet the current MI criteria. OMI is defined as a pathology where there is an acute coronary occlusion or near occlusion with insufficient collateral circulation such that myocardium will imminently infarct without immediate reperfusion. Previous studies found predefined OMI ECG findings to be more sensitive while maintaining specificity. The integration of OMI/NOMI enables for diverse ECG findings to be taken into account when determining reperfusion needs. Investigators have found unique ECG indicators such as hyperacute T waves, J point deviation, etc, that have no room within the current MI guidelines, some of which could be found in the patient in this case report. Still some patients will present with no obvious ECG signs and need to be considered holistically, taking into account significant familial history, symptoms, and relevant biomarkers.^6^,^7^,^8^,

Taken together, this supports the need to continue to revisit current standard of care regarding acute myocardial infarction.

## Supplementary Information







## Figures and Tables

**Figure 3:**