# Supplementary figures and images for: ’Cath’ It Before It’s Too Late: A Case Report of ECG Abnormalities Indicative of Acute Pathology Requiring Immediate Catheterization
Source: J Educ Teach Emerg Med. 2022 Jul 15;7(3):V1–5. doi: 10.21980/J8HW7V (PMC10332696; doi:10.21980/J8HW7V)

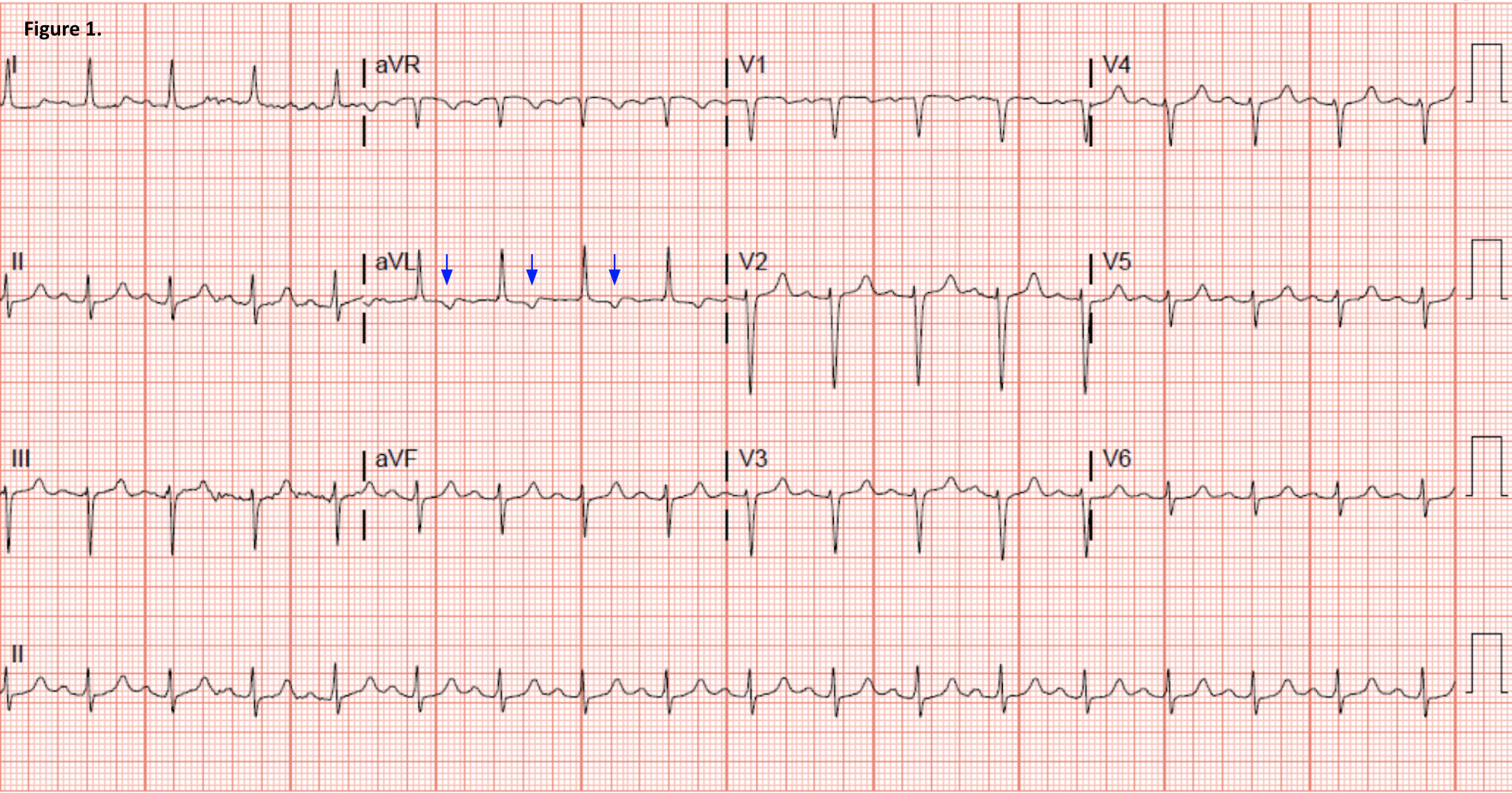

Supplement: Supplementary file 1 [file jetem-7-3-v1-supp1.jpeg]

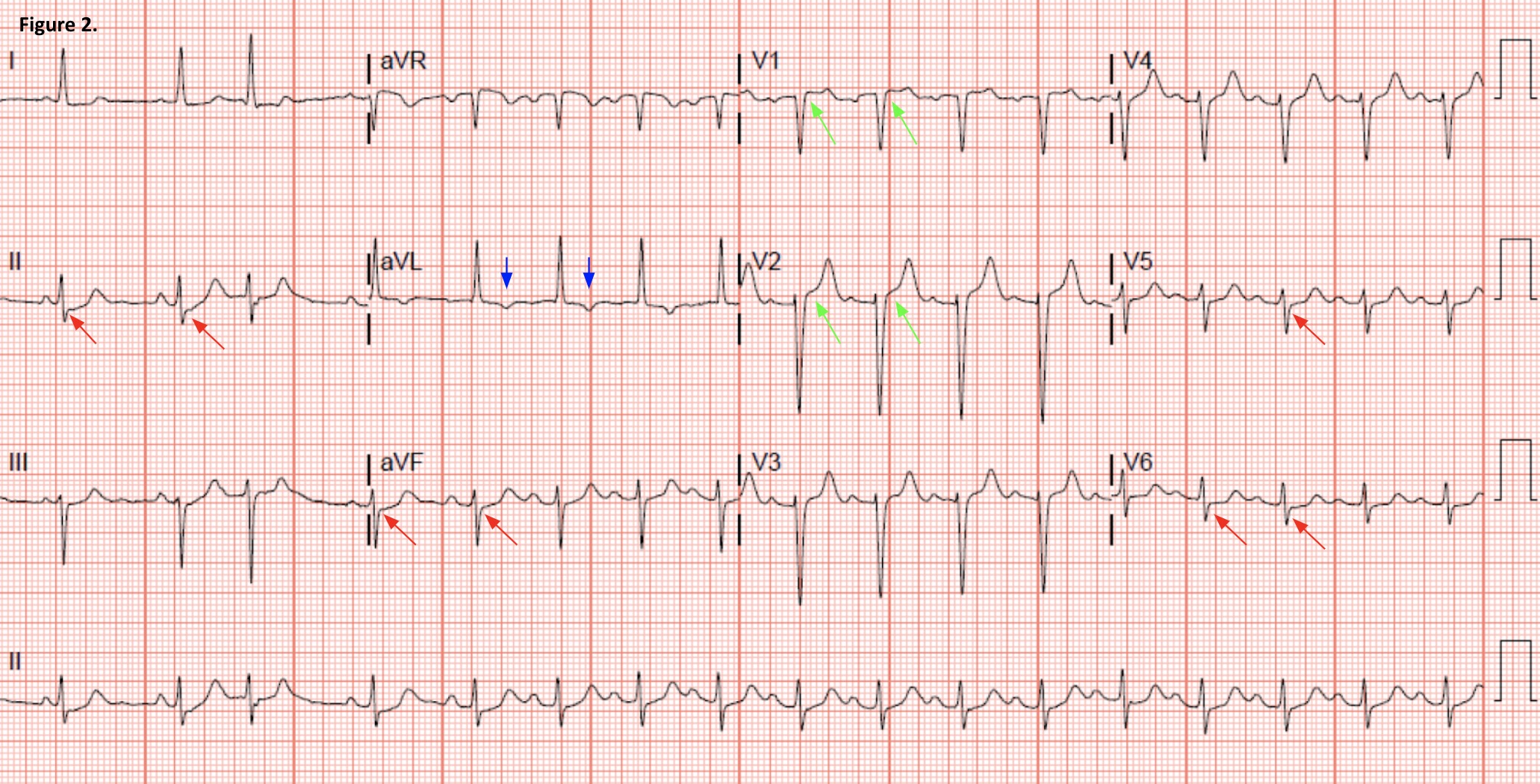

Supplement: Supplementary file 2 [file jetem-7-3-v1-supp2.jpeg]

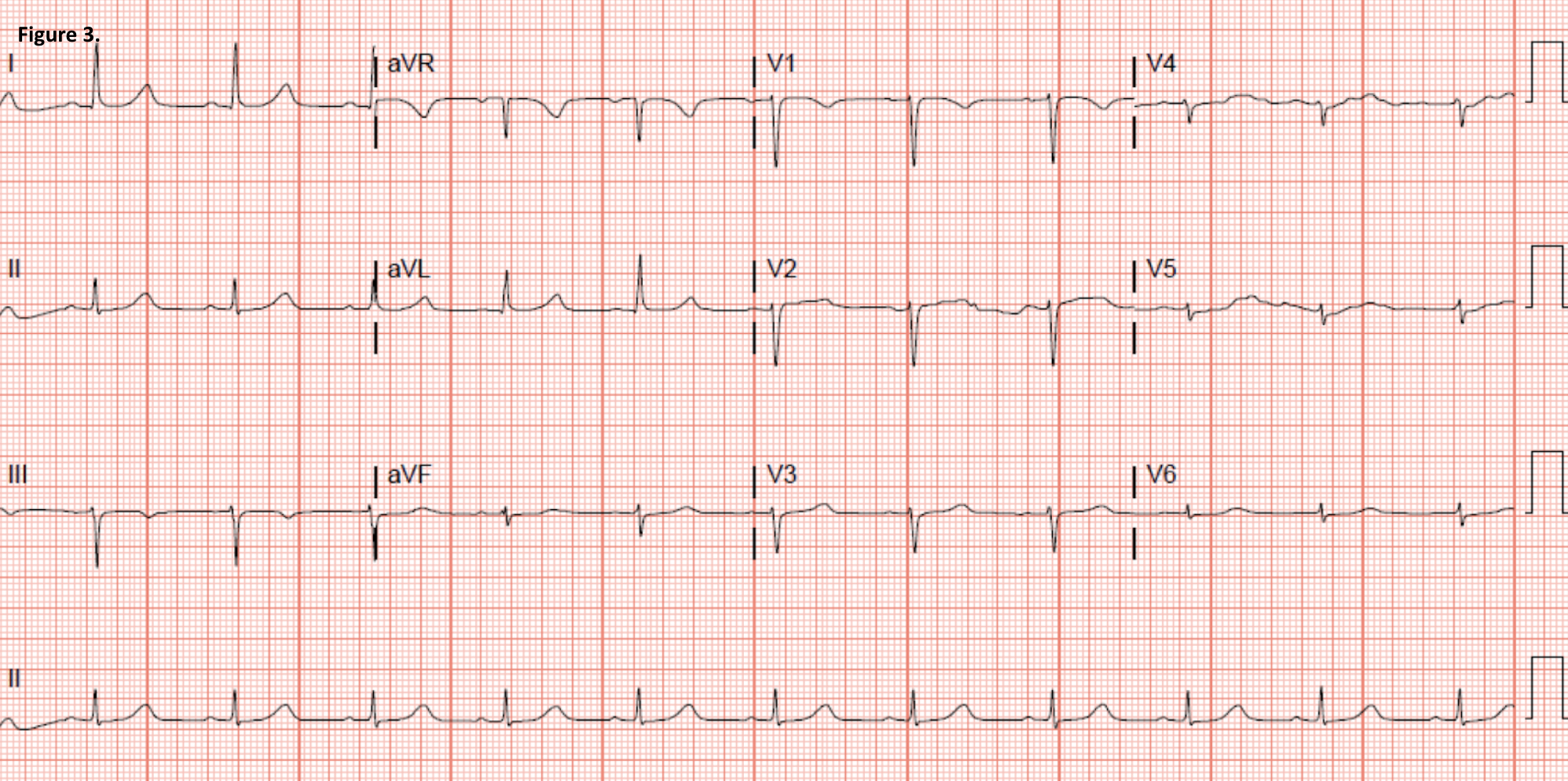

Supplement: Supplementary file 3 [file jetem-7-3-v1-supp3.jpg]
